# Therapeutic effects of saffron and its components on neurodegenerative diseases

**DOI:** 10.1016/j.heliyon.2024.e24334

**Published:** 2024-01-10

**Authors:** Sahar Golpour- Hamedani, Makan Pourmasoumi, Sudiyeh Hejri Zarifi, Gholamreza Askari, Tannaz Jamialahmadi, Mohammad Bagherniya, Amirhossein Sahebkar

**Affiliations:** a Department of Community Nutrition, School of Nutrition and Food Science, Isfahan University of medical science, Iran; b Gastrointestinal & Liver Diseases Research Center, Guilan University of Medical Sciences, Rasht, Iran; c Department of Nutrition, Mashhad University of Medical Sciences, Mashhad, Iran; d Nutrition and Food Security Research Center and Department of Community Nutrition, School of Nutrition and Food Science, Isfahan University of Medical Sciences, Isfahan, Iran; e Anesthesia and Critical Care Research Center, Isfahan University of Medical Sciences, Isfahan, Iran; f Medical Toxicology Research Center, Mashhad University of Medical Sciences, Mashhad, Iran; g Biotechnology Research Center, Pharmaceutical Technology Institute, Mashhad University of Medical Sciences, Mashhad, Iran; h Applied Biomedical Research Center, Mashhad University of Medical Sciences, Mashhad, Iran

**Keywords:** Herbs, Saffron, Saffron compounds, Neurodegenerative disease, Neuroinflammation, Antioxidant

## Abstract

Due to an increase in the number of older people in recent years, neurodegenerative diseases as the most important age-related neurological disorders are considered as a great threat to human health. The treatment strategies for these disorders are symptomatic and there is no known definitive treatment; however, recently, several studies have investigated the effectiveness of some herbs and their components in limiting the progression and treatment of neurodegenerative disorders. In this study, we searched Medline (via PubMed), Scopus, Science Direct, and Google Scholar databases. The keywords used in the search were: saffron [title/abstract] or (saffron compound [title/abstract]) and (neurological disorders [title/abstract]), publication date range (2010–2023), and language (English). After applying inclusion and exclusion criteria, 30 articles remained. Of the 30 articles included in the study, six studies on the treatment of neurodegenerative disorders by saffron and its components were in the clinical trial phase, and 24 studies were in the preclinical phase. Saffron and its compounds can play an important role in inhibiting neuroinflammation and excitotoxic pathways, modulating autophagy and apoptosis, attenuating oxidative damage, and activating defensive antioxidant enzymes, resulting in neuroprotection against neurodegenerative diseases. Therefore, this study aimed to review the studies on the effects of saffron and its compounds on the treatment of neurodegenerative diseases.

## 1 Introduction

Neurodegenerative disorders (NDs) include diseases affecting neurons and gradually lead to neuronal loss [1]. Alzheimer's disease (AD), Parkinson's disease (PD), Huntington's disease (HD), Amyotrophic lateral sclerosis (ALS), and Multiple sclerosis (MS) are the most common neurodegenerative diseases [2]. They are identified by a progressive decrease in sensory-motor, cognitive function, and behavioral abilities, leading to neuronal loss associated with age [2]. Genetics, aging, neuroinflammation, oxidative stress, and excitotoxicity are the most important factors contributing to the occurrence of NDs [[3], [4], [5], [6]]. Neurodegenerative diseases have presented a variety of symptoms, depending on different damages to the nervous system. Changes in mitochondrial function, abnormal accumulations of proteins and proteases, cell damage and death due to oxidative stress, and changes in neuromuscular control with different degrees are some of the characteristics of these diseases [7].

It is expected that the prevalence of neurodegenerative diseases will be augmented by increasing life expectancy in most countries. Neurodegenerative disorders are associated with aging in the elderly, especially those aged 70 and over, with PD and AD as the most common types. Currently, about 50 million people are suffering from dementia worldwide [8], and it is estimated to increase to 130 million people by 2050 [9]. Neurodegenerative diseases affect mental and physiological functions, some cause cognitive dysfunctions, and others affect a person's capability to move and speak [10]. Dementia, slow movements, and cognitive decline are the major symptoms of PD and AD. However, mental disorders including anxiety and depression are secondary complications in neurological diseases that should not be ignored [11].

In general, there is no definitive treatment for neurodegeneration mainly due to the gradual loss of neurons function and neuronal death [12]. Many symptomatic treatments have been developed to reduce perceptual, cognitive, sensory, and motor symptoms to increase people's quality of life to some extent. There are numerous and different age-related changes in the nervous system; therefore, targeting a single change cannot be effective for degenerative disease treatment [10]. Also, there is a complex overlap between brain disorders, and because AD and PD are multifactorial disorders, almost all available drugs can mainly reduce symptoms [11]. Therefore, the use of compounds with multiple biological activities affecting age-related changes in the brain is crucial to limit the progression of the disorder and treat the disease.

Saffron and its compounds have strong biological and antioxidant activities, which have been considered by many researchers for the treatment of neurodegenerative diseases. *Crocus sativus* L, saffron, as a natural additive, is one of the most expensive herbs in the world today. It is herbaceous, and multi-stemmed, which is used as a medicinal herb and spice in many countries. It is cultivated all over the world, especially in Southwest Asia and Mediterranean Europe [13], and due to difficult and manual harvesting and low production volume, it is called red gold. Saffron belongs to the Iridaceae family with many compounds with biological and antioxidant activities. Its secondary metabolites include isoflavonoids, flavonoids, triterpenoids, quinone, and phenolic acids. Active substances in saffron are carotenoids crocin and crocetin, picrocrocin, safranal, and etc [14]. It has small amounts of beta-carotene, sterols, such as campesterol, stigmasterol, and β-sitosterol, as well as linoleic and linolenic acid, ursolic acid, palmitoleic acid, palmitic acid, and oleic acid. Crocins, picrocrocin, crocetin, safranal and flavonoids (quercetin and kaempferol) are the most important compounds of saffron [15].

Pure saffron extract and its compounds have anti-inflammatory, antioxidant, analgesic, antimicrobial, antidepressant, and neuroprotective effects [[16], [17], [18], [19], [20]]. The effectiveness of several saffron compounds on anxiety, depression, and nerve cell disorders has been reported [17,[21], [22], [23]].

Several studies have examined the potential neuroprotective effects of saffron and its bioactive compounds such as crocin, crocetin and safranal. Research suggests saffron may provide benefits in neurodegenerative diseases including Alzheimer's, Parkinson's and Huntington's diseases [24]. In Alzheimer's disease, saffron appears to inhibit formation and accumulation of amyloid beta plaques, which are characteristic Alzheimer's pathology. Saffron has also shown potential to improve memory and cognition in Alzheimer's patients [25]. In Parkinson's models, saffron demonstrates protection of dopaminergic neurons that degenerate in this disease [26]. Saffron extract can reduce oxidative stress, neuroinflammation and improve motor function in Parkinson's models [27].

While the precise mechanisms behind saffron's effects on neurodegenerative diseases are still being investigated, some potential mechanisms including antioxidant activity, anti-inflammatory effects, and anti-apoptotic effects have been proposed based on preclinical and clinical studies [28]. Saffron and its compounds like crocin and crocetin have strong antioxidant properties that can neutralize reactive oxygen species (ROS), which play a major role in neurodegeneration [29]. Additionally, saffron demonstrates anti-inflammatory effects by suppressing production of pro-inflammatory cytokines and inflammation pathways, that are overactivated in these disorders [30]. Saffron also shows anti-apoptotic activity by regulating pathways involved in programmed neuron cell death, a contributor to neurodegeneration. Components like crocin can reduce apoptosis and neuron loss [31]. Moreover, Saffron compounds can modulate serotonin, dopamine, glutamate and other systems involved in neuronal signaling, that are often dysregulated in neurodegenerative diseases [32]. By restoring neurotransmitter balance, saffron may improve neuronal function [32]. Saffron is an accepted antidepressant with fewer side effects compared to antidepressants, such as anxiety, headache, and nausea [33].

Given the effectiveness of saffron in improving many NDs and its low side effects; therefore, this study aimed to investigate the effects of saffron and its compounds on the treatment of neurodegenerative diseases by reviewing the relevant studies.

## 2 Methodology

This process was carried out independently by two reviewers, with the 3rd reviewer clarifying any discrepancies. The collected data were combined and compared for accuracy with the third reviewer. The data collected from the selected studies included: articles containing the two keywords saffron and its compound and neurodegenerative disorders.

### 2.1 Search strategy

After selecting the keywords, they were searched in Medline (via PubMed), Scopus, Science Direct, and Google Scholar databases, using the keywords Saffron [Title/Abstract] OR (saffron compound [Title/Abstract]) AND (neurodegenerative disorders [Title/Abstract]). Filters were applied for the publication date range (2010–2023) and language (English). Then, the full text of the review and irrelevant studies were excluded.

## 3 Results

In this search strategy, about 8000 English language articles were identified. After applying the following restrictions, 30 articles were included in the study: years: 2010–2023, language: English. Of the 30 included studies, six articles referred to clinical trial studies and 24 to preclinical studies. Then reviewing all the included studies, the effect of saffron and its compounds in the treatment of neurodegenerative diseases has been done in two stages. 1. Preclinical studies and 2. Clinical trial studies (Table 1, Table 2).

**Table 1:** Pre-clinical studies investigating the effects of saffron and its compounds on the treatment of neurodegenerative diseases.

Author, Year Country (Reference)	Study design	Intervention/Dosage	Duration	Beneficial effects
Ochiai, 2007 Japan [41]	In vitro study/PC12 Alzheimer's cell line	Crocin (10 μM)	24 h	↑ GSH synthesis
Rafieipour, 2018 Iran [45]	In vitro study/PC12 Alzheimer's cell line	Safranal (2.5 and 5 μM)	120 min	↓ Aβ toxicity
Ghofrani, 2022 Iran [47]	Experimental study/Male rats model of Alzheimer	Crocin (50 mg/kg)	3 weeks	↓ Hippocampal level of ROS ↑ Activity of SOD
Salama, 2020 Egypt [56]	Experimental study/Parkinson mouse model	Crocin (30 mg/kg)	30 days	- Activation of the mTOR pathway ↓ α-synuclein ↓ Apoptosis
Zhang, 2015 China [57]	In vitro study/Pheochromocytoma PC-12 cells	Crocin (0.1, 1, 10, or 100 μM)	0, 3, 6, or 9 h after exposure to MPP+	Endoplasmic reticulum stress and mitochondrial dysfunction inhibition
Ahmad, 2005 India [58]	Experimental study/Male Wistar rats, model of Parkinson	Crocetin (25, 50 and 75 μg/kg	7 days	↓ Thiobarbituric acid reactive substance (TBARS)
Mohammadzadeh, 2018 Iran [59]	Experimental study/Rat model of Parkinson	Crocin (10, 20, or 40 mg/kg/day)	28 days	↓ Lipid peroxidation - Improvement of neurobehavioral impairments
Purushothuman, 2013 Australia [60]	Experimental study/Mouse model of Parkinson	Saffron (0.01 % w/v)	5 days	Dopaminergic cells retina protection from Parkinsonian insult
Hatami, 2015 Iran [61]	Experimental study/Rat model of Parkinson	Saffron extract (5 and 10 μg/rat)	5 days	Spatial memory improvement
Pan, 2017 China [62]	In vitro study/Primary dopaminergic cells	Safranal (10, 15, 20, and 50 μg/ml)	4 h	↓ ROS generation ↓ Apoptosis ↑ Nrf2
Fotoohi, 2020 Italy [69]	Experimental study/Male Wistar rats with HD	Safranal (0.75, 1.5 and 3 mg/kg)	2 weeks	Reduced the changes in the number of vacuous chewing movements and locomotor activity
Zhang, 2020 China [70]	Experimental study/Male Sprague-Dawley rats	Crocin (25, 50, and 100 mg/kg)	3 days	↑ Antiapoptotic protein Bcl-2 ↑ SOD, GSH, and GSHPx expression level ↓ MDA ↓ Bax and caspase-3 expression level
Krishnaswamy, 2020 India [71]	Experimental study/Male Wistar rats	Crocetin (50 and 150 mg/kg)	45 days	↑ Acetylcholine content - Acetylcholinesterase inhibition - Improved mitochondrial function
Salem, 2022 Beirut [89]	Experimental study/Mouse model of brain injury	Saffron (50 mg/kg) and Crocin (30 mg/kg)	14 days	↓ TNF-α ↓ IFN-γ ↓ MPO ↓ MDA
Ghaffari, 2015 Iran [96]	Experimental study/Rats model of MS	Ethanolic extract of saffron (5 μg/rat and 10 μg/rat)	1 week	↑ Antioxidant enzymes GPx and SOD
Papandreou, 2011 Greece [99]	In vitro- in vivo study/Male Balb-c mice for animal phase and human neuroblastoma SH-SY5Y cell line for in vitro study	Saffron (60 mg/kg BW)/Saffron (1–250_μg/mL) and safranal (1–125_μM)	6 days/18 h	- Learning and memory improvement ↓ Lipid peroxidation ↓ Caspase-3 activity ↓ ROS production ↑ Cell viability
Ghaffari, 2013 Iran [101]	Experimental study/Male rats model of MS	Saffron extract (5 and 10 μg/rat)	3 days	↑ Spatial memory ↓ MDA
Tashakori, 2023 Iran [102]	Experimental study/Mouse model of MS	Crocin (100 mg/kg)	5 weeks	↑ SOD ↑ GPx ↑ Total antioxidant status
Vakili, 2011 Iran [107]	Experimental study/Rat model of cerebral ischemia	Saffron (100 mg/kg)	1 day	↓ MDA ↑ The activity of superoxide dismutase
Zhong, 2020 China [108]	Experimental study/Rats with ischemic brain damage	Saffron extract (30, 100, 300 mg/kg)	46 days	↓ IL-6 and IL-1β ↑ IL-10 ↓ Astrogliosis and glial scar
Abdel-Rahman, 2020 Egypt [110]	Experimental study/Rat model of cerebral ischemia	Saffron extract (100 or 200 mg/kg)	3 weeks	↓ Lipid peroxidation, ↓ NO content and brain natriuretic peptide (BNP) ↓ Caspase-3 and Bax protein expression ↓ Apoptosis
Chang, 2014 Taiwan [79]	in vitro study/Spinocerebellar ataxia cell	Geniposide, crocin, and genipin (50–100 nM)	8 h	↓ PolyQ accumulation ↓ ROS
Fernández, 2012 Spain [114]	Experimental study/Rat model of retinitis pigmentosa	Safranal (400 mg/kg)	1 week	- Preservation of the capillary network ↓ cell degeneration of photoreceptor
Fernández, 2019 Spain [115]	Experimental study/Mouse model of glaucoma	Saffron extract (60 mg/kg)	1 week	↓ Microglia ↓ Death of retinal ganglion cells

**Table 2:** Clinical trial studies investigating the effects of saffron and its compounds on the treatment of neurodegenerative diseases.

Author, Year Country (Reference)	Population	Intervention/Dosage	Duration	Beneficial effects
**Asadollahi, 2019** **Iran** [109]	Stroke patients	Saffron (200 mg/day)	3 months	↓ Severity of stroke ↓Serum NSE and s100 levels
**Ghiasian, 2019** **Iran** [100]	MS patients	Crocin (30 mg/day)	28 days	↓ Lipid peroxidation ↓ DNA damage ↓ Tumor necrosis factor-alpha ↓ Interleukin 17
**Ghasemi, 2020** **Iran** [98]	MS patients	Saffron (500 mg, three times a day)	12 months	↓ serum MMP-9 levels ↑ TIMP-1
**Broadhead, 2019** **Australia** [111]	AMD patients	Saffron (20 mg/day)	3 months	Visual function improvement
**Lashay, 2016** **Iran** [112]	AMD patients	Saffron (30 mg/d)	6 months	Improvement in retinal function in patients with AMD
**Gudarzi, 2022** **Iran** [90]	Ischemic strokes patients	Saffron (400 mg/day)	4 days	↑ GSH ↑ TAC ↓ MDA

## 4 Pre-clinical studies

### 4.1 Alzheimer's disease (AD)

AD is associated with the formation of nerve fiber twists and amyloid plaques, which can cause pathological disturbances in the brain, followed by the loss of learning and memory abilities [34]. AD is caused by amyloid-β (Aβ) deposition and is strongly correlated to oxidative stress. In the context of Alzheimer's disease, synaptic dysfunction is an early event that disrupts neuronal communication. Amyloid Precursor Protein (APP) is enzymatically cleaved to produce amyloid-beta (Aβ) peptides, which can aggregate to form oligomeric Aβ (OAβ) and eventually Aβ plaques. These plaques are a hallmark of Alzheimer's disease, contributing to neuronal damage and the clinical symptoms of dementia [35]. The signaling process shows that the aggregation of Aβ leads to an increase in caspase 3 activity, an enzyme involved in the execution phase of cell apoptosis, indicating that the accumulation of Aβ is associated with neuronal death [36]. Accompanying this is a decrease in ATP levels, the energy currency of the cell, implying that Aβ accumulation disrupts cellular energy homeostasis [37]. Moreover, an increase in reactive oxygen species suggests that Aβ might contribute to oxidative stress, which can further damage neurons in Alzheimer's disease [38]. Impaired glutamate recycling is another key aspect in Alzheimer's disease. Glutamate is a vital neurotransmitter for normal synaptic function, and its dysregulation is implicated in excitotoxicity and neuronal death in Alzheimer's disease [39].

**Fig. 1:**
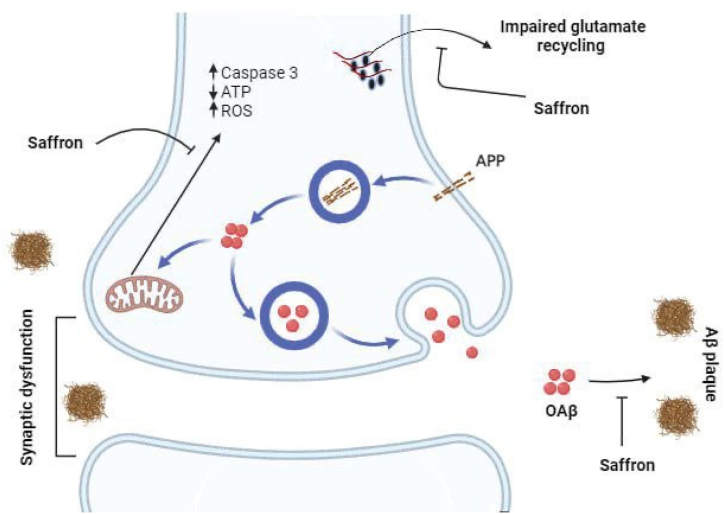
The process related to the pathophysiology of Alzheimer's disease and the potential intervention by saffron.

Saffron is a potential therapeutic agent that can modulate this pathological signaling process in Alzheimer's disease. Saffron is shown to potentially inhibit the formation of Aβ plaques, suggesting it might interfere with the cleavage of APP or the aggregation of Aβ peptides [40]. Moreover, saffron is linked to the normalization of impaired glutamate recycling, indicating its possible role in protecting synaptic function [40] (Fig. 1).

Alzheimer's disease is the most common cause of dementia, and its prevention and treatment strategies should be highly considered [8]. Given the strong relationship between AD and inflammation and oxidative stress, and the anti-inflammatory effects of saffron through flavonoids, tannins, saponins, and crocin; therefore, saffron and its compounds seem to play an important role in AD treatment [41]. The production of inflammatory metabolites (especially Aβ) increases the inflammatory process and given that free radicals are mediators of Aβ-induced neurotoxicity in the development of AD; therefore, treatment with saffron extract can prevent Aβ-related oxidation reactions [42]. Crocin and crocetin possess neuroprotective effects through a reduction in oxidative stress, neuroinflammation, blood-brain barrier (BBB) damage, and nerve cell apoptosis. Also, they exert a neuroprotection effect through a decrease in Aβ accumulation and synaptic loss [43,44]. Rafieipour et al. reported that saffron exerts its neuroprotective effects through the mitogen-activated protein kinase (MAPK) and phosphatidylinositol 3-kinase (PI3K) pathways, which are involved in cell survival of neuronal tissues [45]. Tahvili et al. investigated the therapeutic effects of saffron on AD mice and demonstrated the protective effects of saffron on the nervous system via decreased inflammation, however, the result was not statistically significant [46]. Considering a relationship between oxidative stress and AD, Ochiai et al. showed that flavonoids, tannins, saponins, and crocins in saffron could reduce inflammation and oxidative stress. In their study, crucin via increased expression of γ-glutamylcysteinyl synthase (γ-GCS) led to glutathione (GSH) synthesis, as an antioxidant agent [41]. In this regard, Ghofrani et al. also demonstrated the protective effects of crocin on conditional learning and memory deficits. In addition, crocin significantly reduced hippocampal reactive oxygen species levels and enhance superoxide dismutase (SOD) activity, as well as attenuating apoptotic factors and DNA fragmentation [47]. Several studies have shown that crocin and crocetin exert neuroprotection by a reduction in oxidative stress, invasion and phosphorylated thiol formation, synaptic loss, and apoptosis of nerve cells [43,48]. Depression and anxiety are consequences of AD, which are extremely traumatic and challenging in the long term. Several studies have indicated the effects of saffron compounds on depression and anxiety in AD patients [17,21].

Depression and anxiety are among the common diseases associated with AD, affecting the quality of life of these patients. Depression and anxiety accelerate the progress of AD and lead to increased mortality of AD patients. Given the antidepressant effects of saffron [49]; therefore, it can be considered a suitable therapeutic option [50,51], improving the life quality of AD patients [52,53].

### 4.2 Parkinson's disease

PD, which is the second most common type of neurodegenerative disease after AD, can be caused due to insufficient defense against the oxidative stress of dopamine-secreting neurons, associated with the basic dysfunction of dopamine because of the presence of Lewy bodies. In PD, the lack of dopaminergic neuron function is observed in the substantia nigra of the limbic system [54]. PD symptoms include tremors, slowness of movements, muscle stiffness, imbalance, and loss of involuntary movements [55]. Salama et al. investigated the effect of saffron on the regulating cell cycle pathway, PI3K/Akt/mTOR, in a PD mouse model. They showed that at the cellular level, crocin significantly stimulated the PI3K/Akt pathway and increased the levels of mTOR, leading to a decrease in apoptotic factors including glycogen synthase kinase-3β (GSK-3β), forkhead box transcription factor of the O class (FoxO3a), and the downstream caspase-9. Therefore, a decrease was observed in neurodegeneration through increasing tyrosine hydroxylase (TH) and dopamine (DA). Also, in the study, crocin led to an increase in the microRNA-7 (miRNA-7) and miRNA-221 expression, resulting in Akt/mTOR activation. Regarding the mTOR pathway, crocin relieves apoptosis and α-synuclein formation [56].

Regarding other protective effects of saffron, Zhang et al. showed that saffron and its constituents exerted pheochromocytoma (PC-12) cells protection against the damage caused by the neurotoxin 1-methyl-4-phenylpyridinium (MPP^+^) by inhibiting endoplasmic reticulum stress and mitochondrial dysfunction [57]. Ahmad et al. demonstrated that crocin had neuroprotective effects in PD-induced mouse models with hydroxydopamine by reducing oxidative stress [58]. Mohammadzadeh et al. confirmed this effect and showed that crocin improved movement defects and reduced inflammatory cytokines in a malathion-induced PD model [59]. Purushothuman et al. showed the neuroprotective effects of saffron on dopaminergic HeLa cells in rats and confirmed the relationship between the interactions of dopaminergic and cholinergic systems [60]. Hatami et al. investigated the improvement of spatial memory by saffron extract in Parkinsonian rats and showed that pretreatment with saffron extract in PD rats led to a significant improvement in spatial memory [61]. Pan et al. demonstrated the protective effects of saffron on primary dopaminergic cells against cell death by promoting cytoprotective responses to oxidative stress through the increased expression of nuclear factor erythroid 2–related factor 2 (Nrf2), as a regulator of cellular resistance to oxidants, in rotenone-induced PD models [62]. Also, Inoue et al. reported the neuroprotective effects of crocin and crocetin through inhibition of the major constituent of Lewy bodies, and α –synuclein accumulation [63]. Generally, saffron and its components may have potential beneficial effects in managing Parkinson's disease by protecting neurons from oxidative stress, reduce inflammation, and improve motor function. However, further research is needed to stablish its efficacy, and safety in clinical studies.

### 4.3 Huntington's disease

HD is a progressive neurodegenerative disorder caused by the repetition of cytosine-adenine-guanine (CAG) trinucleotide in the huntingtin (HTT) gene [64]. HD is characterized by physical, psychological, and behavioral changes, leading to motor and cognitive dysfunction [65]. It is also associated with a progressive loss of neurons, active astrocytes, and brain structure (e.g., putamen and caudate nucleus) [66]. Aβ-related diseases like HD, are caused by the accumulation of intracellular and/or extracellular proteins in fibrillar deposits [67]. So far, more than 25 different proteins and peptides have been found to form Aβ aggregates in humans, and almost all proteins can form Aβ fibrils under special conditions [68].

Regarding the effects of saffron and its compounds on the progression of HD, Fotoohi et al. showed that saffron significantly reduced the changes caused by 3-Nitropropionic acid (3-NP) in body weight, functional activity, and the number of vacuum chewing movements (VCMs) in mice with HD. In addition, the assessment of brain tissue showed that saffron could prevent an increase in nitrite oxide and malondialdehyde (MDA) levels and a decrease in SOD, glutathione (GSH), and catalase activity caused by 3-NP [69]. Also, Zhang et al. reported increased levels of SOD, GSH, and GSHPx expression and decreased levels of MDA and glutathione disulfide (GSSG) after treatment with crocin. Also, the expression levels of the anti-apoptotic B-cell lymphoma 2 (Bcl-2) family proteins increased, along with decreased levels of apoptosis regulators Bax and caspase-3 expression in a dose-dependent manner. It seems that the cognitive protection of crocin against acute high-altitude hypoxia is through mitochondrial biosynthesis and oxidative stress improvement, as well as neuronal apoptosis reduction [70]. It seems that the positive effects of the saffron extract on HD are dose-dependent. In this regard, Krishnaswamy compared the effects of supplementation with crocin 50 and 150 mg/kg on the cerebral cortex and showed that crocin supplementation significantly reversed age-related macromolecular damage, decreased endogenous antioxidants, and increased intracellular calcium concentration. Also, crocin at a dose of 150 mg/kg significantly improved acetylcholine levels through acetylcholinesterase inhibition. In addition, crocin at 150 mg/kg significantly improved mitochondrial function, evidenced by citrate synthase and cytochrome C oxidase enzyme activity [71]. Further well-designed clinical trials are needed to validate the potential benefits of saffron in HD and to determine the optimal dosage, treatment duration, and safety profile.

### 4.4 Ataxia

Ataxia is a movement disorder, which can be hereditary, acquired, or sporadic, and also it is a clinically and genetically heterogeneous group of neurodegenerative diseases, resulting in cerebellum dysfunction or impaired communication between the cerebellum and other CNS areas [72]. It is associated with pyramidal signs, movement disorder, convulsions, peripheral neuropathy, and cognitive impairment. In addition, patients with ataxia often have many other problems, including instability in standing and walking, dysarthria, nystagmus, dysmetria, and clumsiness [73]. To date, there is no effective treatment for ataxia, and current treatments focus only on symptomatic management [72]. Therefore, using effective combinations and substances to manage its symptoms is of great importance.

Mitochondrial dysfunction was found in brain samples of ataxia patients, it, therefore, seems that the resulting ROS leads to cumulative oxidative-nitrative damage [74]. In patients with autosomal dominant cerebellar ataxias (ADCA), a decrease in the levels of GSH and other thiols could increase their sensitivity to oxidative damage [75,76], resulting in DNA damage caused by ROS and cell death and consequently, movement disorders [77]. Moreover, ROS led to protein accumulation and more ROS production [78]. Therefore, using antioxidants can be a therapeutic strategy against ataxia. Experimental studies have shown that in ataxic cells polyglutamine stretch (PolyQ) aggregates inhibit the activation of nuclear factor erythroid-related factor 2 (NRF2), as an antioxidant marker. Chang et al. showed that crocin along with other active antioxidants could prevent the accumulation of polyQ in spinocerebellar ataxia cells. Also, crocin could reverse decreased expression of NRF2 and antioxidant enzymes, including NAD(P)H quinone dehydrogenase 1 (NQO1), and glutathione *S*-transferase P 1 (GSTP1), and suppress the regeneration of ROS and polyQ accumulation in neurally differentiated cells, which all lead to endogenous defense against ataxic damage [79]. These studies suggest that saffron may have neuroprotective properties and could potentially play a role in promoting brain health. Nevertheless, more research is needed to determine the specific effects of saffron on ataxia and its underlying mechanisms.

## 5 Clinical trial studies

### 5.1 Amyotrophic lateral sclerosis (ALS)

ALS, as a motor neuron dysfunction, causes progressive and irreparable damage to the central and peripheral nervous systems. ALS is correlated with both upper and lower motor neuron symptoms [80], leading to gradual loss of muscle function, especially striated muscles, due to their progressive atrophy. It also can weaken the muscles; therefore, the patients gradually develop general paralysis, and they usually do not have a long-life span [81]. ALS can be developed due to DNA damage, neuro-inflammation and oxidative stress, apoptosis, mitochondrial mutations, accumulation of proteins, such as prion, α-synuclein, Aβ and tau, as well as, toxic intracellular proteins accumulation due to changes in the degradation pathway, and impaired biochemical homeostasis in the central nervous system (CNS) [82]. Also, gene mutations of the superoxide dismutase 1 gene (SOD1) are associated with ALS [83]. Therefore, antioxidants like saffron can play an important role in preventing and limiting the disease.

Eom et al. showed antioxidants might be effective in delaying ALS progression and increasing the life expectancy of patients [84]. Cellular oxidation processes play a role in ALS, and antioxidant agents are promising for the treatment of ALS [[85], [86], [87]]. The isolation of secondary metabolites from natural sources and semi-synthetic derivatives should be considered to develop therapeutic strategies [88]. Salem et al. assessed the antioxidant impacts of saffron and crocin on brain damage and showed that both saffron and crocin decreased the TNF alpha (TNF-α), interferon-gamma (IFN-γ), and MDA levels, and myeloperoxidase (MPO) activity, which led to significant improvement in physical, neurological and motor functions [89]. Skhalyakhov et al. using bioinformatic pathway enrichment analysis showed that saffron, like other plant-derived chemicals, had a hermetic stimulating effect beyond its strong antioxidant capacity [86]. Gudarzi et al. also demonstrated the therapeutic effects of saffron extract for NDs in humans by increasing antioxidant enzyme activity such as SOD and glutathione peroxidase (GPx), GSH levels, and total antioxidant capacity (TAC) and reducing MDA levels in stroke patients [90]. However, the review of studies showed a limited number of investigations on the effects of saffron on ALS.

### 5.2 Multiple sclerosis

MS, as the CNS chronic inflammatory disease, is associated with the demyelination of neurons causes damage to axons and myelin, and affects sensory, motor, and cognitive functions [91]. The MS onset usually occurs between the ages of 20 and 40 years, and women are twice as likely to have MS as men [92]. It is the third cause of disability among adults [93]. Inflammation, demyelination, oxidative stress, and axonal and repair mechanism damage cause MS [94]. Also, the parts of the nervous system responsible for communication are disrupted, leading to signs and symptoms, including physical and psychological problems, and in some cases, psychiatric disorders. Unlike other chronic wasting diseases, MS occurs mostly in young people, especially in women. It is a heterogeneous disease with mild to severe severity and in some cases, causes paralysis [92]. Oxidative stress plays an influential role in the formation and progression of MS lesions. Profound changes in mitochondrial respiratory chain proteins and mitochondrial DNA deletions have been observed in neurons with active lesions in the advanced stages of MS [95].

In this regard, Ghaffari et al. assessed the effect of saffron ethanol extract on oxidative stress indices in MS and found that it significantly enhanced the activity of GPx and SOD enzymes [96]. Ghaffari et al. also showed a significant modulation of the total reactive antioxidant capacity, antioxidant enzyme activity, and lipid peroxidation in hippocampal homogenates after one week of treatment with saffron extract [97]. Ghasemi et al. showed that the 12-month treatment with 500 mg saffron tablets three times a day led to the regulation of T cells movement to CNS by reducing the serum levels of matrix metalloproteinases 9 (MMP-9) and increasing the serum levels of tissue inhibitor of metalloproteinases-1 (TIMP-1) in MS patients [98]. Papandreou et al. reported a significant increase in the antioxidant activity in the brain of old rats after seven days of intraperitoneal injection of saffron. Also, human neuroblastoma cells treated with saffron showed decreased free radicals production, along with increased cell viability [99]. Furthermore, Ghiasian et al. investigated the effects of saffron supplementation on MS patients and reported a significant reduction in DNA damage, lipid peroxidation, and tumor necrosis factor-alpha (TNFα), which are all pathogenic factors in MS. A significant increase was also observed in serum TAC levels in treated patients with crocin [100]. Ghaffari et al. also evaluated the effectiveness of alcoholic saffron extract (three-day injection) on spatial memory and lipid peroxidation in the hippocampus of mice with MS and demonstrated an improvement in spatial memory and a significant decrease in MDA levels in the saffron group [101]. Regarding functional disorders, Tashakori et al. showed the effects of crocin on the angular movements of the hind leg, keeping the back and front limbs stable, stability in standing, and grip strength in mice with MS, indicating the protective effects of saffron compounds on movement disorders caused by MS [102]. It's worth noting that research on the potential effects of saffron in the context of MS is limited. While saffron's antioxidant and anti-inflammatory properties can exert protective effects, more rigorous scientific studies are needed to determine its specific effects on MS and its potential role in the management of the condition.

### 5.3 Cerebral ischemia

The impaired blood flow to the brain following a stroke leads to a decrease in the concentration of oxygen and metabolic substances in the brain's ischemic areas, resulting in their undetectable levels within a few minutes [103]. This decrease causes a disruption in the function of mitochondria and the production of free radicals. However, improving blood flow after a stroke causes an increase in the production of pro-oxidants, damage to mitochondria, the extrusion of mitochondrial contents, and the acceleration of free radicals production [104]. Both decreased antioxidant enzyme capacity and increased free radical levels lead to cell damage, followed by cell death. Free radicals cause the formation, expansion, and consequently edema and secondary brain injury after stroke [104,105].

Using strong antioxidants may protect the brain against the damage caused by oxidants in cerebral stroke to some extent and reduce neuronal death [106]. Vakili et al. showed that saffron reduced the volume of cerebral lesions by 77 % and cerebral edema by 60 % in oxidative brain ischemia. Likewise, saffron significantly reduced MDA levels and increased SOD and GPx activity in the ischemic tissue of the cerebral cortex [107]. Zhong et al. also showed that the treatment of rats with ischemic brain damage by different doses of saffron improved neurological deficits, spontaneous brain activity, and anxiety disorders like cognitive impairment. Also, saffron reduced the infarct volume, reduced the number of active astrocytes and the thickness of the glial scar in the ischemic area, and increased the neuron density in the cortex in the ischemic border region. In addition, they found a decrease in IL-6 and IL-1β levels and an increase in IL-10 levels in the ischemic area. Therefore, saffron showed neuroprotective properties on delayed cerebral ischemia, associated with a reduction in astrogliosis and formation of glial scar after ischemic injury [108]. Asadollahi et al. evaluated the effects of daily saffron intake (200 mg) on ischemic patients and found lower stroke severity in the intervention group compared to controls, and the serum levels of neuron-specific enolase (NSE) and S100, which are indicators of ischemic severity, significantly decreased. Also, the brain-derived neurotrophic factor (BDNF) levels in the saffron group increased, and the average Barthel score, which indicates functional independence, in the saffron group was significantly higher than that in the control group after a three-month follow-up [109].

Abdel-Rahman et al. assessed the effects of saffron on cerebral ischemia in a mouse model and showed that saffron could significantly reduce lipid peroxidation, nitric oxide (NO) levels, and brain natriuretic peptide (BNP), besides a decrease in the expression of caspase-3 and Bax protein, consequently a significant reduction in apoptotic nerve cells. Saffron also regulated the expression of vascular endothelial growth factor (VEGF) in the ischemic area, leading to neuroprotection [110]. It's important to note that while the findings from preclinical and clinical studies are promising, further research is needed to determine the efficacy and safety of saffron in human subjects with cerebral ischemia. Further clinical trials are necessary to fully understand the potential of saffron in the prevention and treatment of cerebral ischemia.

### 5.4 Retinal neurodegenerative diseases

The purinergic receptor P2X7 with physiological and pathological functions is widely expressed in the nervous system. The P2X7 receptors in the retina are involved in the progression of retinal neurodegenerative disorders, such as retinitis pigmentosa and age-related macular degeneration (AMD). Broadhead et al. examined the effects of saffron on AMD patients and showed that the saffron supplementation (20 mg per day) for at least three months improved visual performance, which might be related to its antioxidant properties [111]. Lashay et al. showed that daily supplementation of AMD patients with saffron (30 mg) significantly improved their retinal function and electroretinography (ERG) results after three months of treatment [112].

Piccardi et al. showed the effectiveness of saffron in the viability of rat primary retinal cells and photoreceptor-derived 661W cells exposed to ATP and reducing the intracellular calcium levels in 661W cells [113]. Fernández et al. investigated the effects of safranal present in saffron on retinal degeneration in a mouse model and showed that safranal preserved the morphology and number of photoreceptors and electroretinographic recordings (a- and b-waves) in photopic and scotopic conditions compared to the control group. Also, the capillary network was maintained and cell degeneration of photoreceptors decreased in animals treated with safranal [114].

Glaucoma as a neurodegenerative disease is induced by the retinal ganglion cells (RGCs) loss, in which the activated microglia can cause RGC death, indicating the important role of the anti-inflammatory and neuroprotective agents in reducing the progression of the disease. Fernández et al. evaluated the effects of saffron extract in a mouse model of glaucoma. Saffron reduced microglion numbers and their morphological indications including soma size and process retraction. Treatment with saffron extract also reversed laser-induced ocular hypertension (OHT) [115]. In summary, saffron shows promise as a potential complementary approach for retinal neurodegenerative diseases. Its antioxidant and anti-inflammatory properties may help protect retinal cells and improve visual function. However, further research is needed to establish its efficacy and optimal dosage.

The results of these two stages showed that saffron and its compounds have the potential to create a protective effect by targeting different signaling pathways such as neuroinflammation, activation of microglia, excitability toxicity, regulation of autophagy, inhibition of apoptosis, and activation of antioxidant enzymes. Nerves are in neurodegenerative diseases. In addition, saffron's ability to reduce oxidative damage suggests that it may be useful in slowing the progression of neurological diseases such as Alzheimer's, Parkinson's, ALS, Huntington's, cerebral ischemia, MS, retinal neurodegenerative diseases, ataxia, and cerebral ischemia.

## 6 Discussion

Neurodegenerative disorders, including AD, PD, ALS, HD, cerebral ischemia, and MS, have specific etiologies and various types of pathology. Oxidative stress as a common characteristic of NDs occurs due to increased production of ROS during the development of NDs. The brain represents the largest source of energy consumption and is a rich source of fat, making it susceptible to oxidative stress and extreme production of ROS. Besides, the high content of polyunsaturated fatty acids in neuronal membranes makes them highly sensitive to ROS; thus, oxidative stress chemically alters bimolecular components, resulting in NDs. In addition, the pathological state of NDs includes the increased aggregation of proteins, leading to chronic inflammation of the nervous system. Therefore, the accumulation of toxic proteins, oxidative stress, neuroinflammation, and excitotoxic pathways, are the main pathological factors contributing to the development of neurodegenerative diseases [116].

**Fig. 2:**
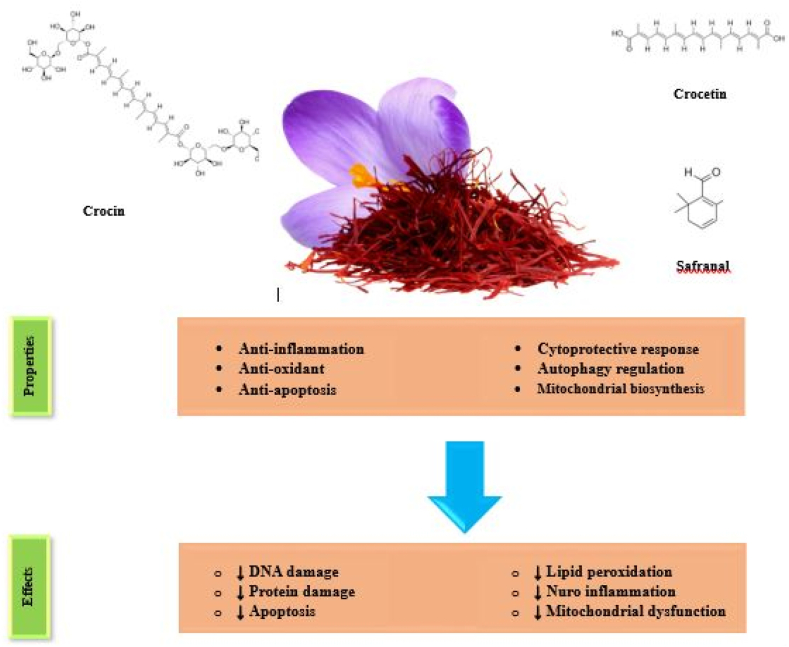
Potential effects of saffron and its constituents (crocin, crocetin, and safranal) on neurodegenerative diseases.

The results of the present study demonstrated that saffron and its compounds might exert neuroprotection in neurodegenerative diseases by inhibiting neuroinflammation, microglial activation, excitotoxic pathway, autophagy regulation, the inhibition of apoptosis, activation of antioxidant enzymes, and reducing oxidative damage. The main potential effects of saffron and its major constituents (crocin, crocetin, and safranal) on neurodegenerative diseases are summarized in Fig. 2.

The production of inflammatory metabolites increases inflammation (especially the production of Aβ), and free radicals are mediators of Aβ-induced neurotoxicity in the development of neurodegenerative diseases. Indeed, Aβ increases the production of free radicals and lipid oxidation in nerve cells, leading to cell death; therefore, treatment with saffron extract as an inhibitor of oxidative stress induced by Aβ can be effective in nervous improvement [46]. Samarghandian et al. examined the antioxidant effects of saffron in old age and showed a decrease in the levels of MDA and NO in the hippocampus and an increase in the antioxidant status of nerve cells [117]. Saffron and its bioactive compounds have different therapeutic functions in physical and psychological disorders, including mental disorders, neurological diseases, cardiovascular diseases, cancer, and diabetes.

Saffron can prevent oxidative damage by a reduction in the concentration of endogenous ROS and a reduction in the production of pro-inflammatory biomarkers [118,119]. Crocin can improve cognitive disorders to some extent by targeting hippocampal apoptosis and oxidative stress [47]. Oral administration of crocin significantly reversed age-related oxidative stress and neuroinflammatory markers and modulated oxidative stress markers by scavenging ROS and free radicals [96]. Fotoohi et al. showed that saffron prevented HD in an animal model of HD, which might be attributed to its modulating effect on oxidative balance [69]. Tashakori et al. confirmed the protective effect of crocin against MS [102]. Also, saffron extract improved learning and memory disorders and the impairment of oxidative stress parameters in the hippocampus of patients with MS [97]. Saffron exerted neuroprotective effects on delayed cerebral ischemia and could help prevent and control the severity of the stroke consequences [108,120]. A limited number of studies were found on the effectiveness of saffron and its components on ataxia. Given the antioxidant effects of saffron and crocin; therefore, crocin might be effective in the treatment of ataxia by decreasing the accumulation of PolyQ. However, more studies are needed to confirm the effectiveness of saffron on other cellular aspects of ataxia.

Considering the effects of saffron on retinal neurodegenerative diseases, our results indicated the effectiveness of saffron and its components on the progression of neurodegenerative diseases. Saffron and its compounds slow the degeneration of photoreceptors, improve the loss of retinal function and vascular network disorder, and can potentially be useful in slowing retinal degeneration [114]. Studies investigating the effectiveness of different doses of saffron and its compounds on the progression of neurodegenerative diseases showed that saffron can be an appropriate option to slow the progression of neurodegenerative diseases due to its strong antioxidant effects. It should be noted that many relevant studies have used different doses and treatment periods, which could be considered the most important limitation of the present study along with the lack of follow-up periods to make a definitive conclusion. It is therefore recommended that cohort studies and large clinical trials with standard doses and the same treatment periods be considered to assess the effectiveness of saffron compounds on neurodegenerative diseases to generalize the results.

## 7 Conclusion

Saffron compounds might improve antioxidant defense and oxidative stress, and reduce Aβ deposits as the most important mechanism of the progression of neurodegenerative diseases. However, limited information is available to make any suggestion regarding the effect of saffron on all disorders. Trials with an adaptive design and providing exclusive results are needed to confirm the effects of saffron and its compounds to help decide on the effectiveness of saffron supplements in neurodegenerative diseases.

## Funding

None.

## Data availability

There is no raw data associated with this review article.

## CRediT authorship contribution statement

**Sahar Golpour- Hamedani:** Writing - original draft, Conceptualization. **Makan Pourmasoumi:** Writing - review & editing. **Sudiyeh Hejri Zarifi:** Writing - review & editing. **Gholamreza Askari:** Writing - review & editing. **Tannaz Jamialahmadi:** Writing - review & editing. **Mohammad Bagherniya:** Writing - review & editing, Conceptualization. **Amirhossein Sahebkar:** Writing - review & editing, Conceptualization.

## Declaration of competing interest

The authors declare that they have no known competing financial interests or personal relationships that could have appeared to influence the work reported in this paper.
